# Auto-scaling LLM-based multi-agent systems through dynamic integration of agents

**DOI:** 10.3389/frai.2025.1638227

**Published:** 2025-09-12

**Authors:** Ravindu Perera, Anuradha Basnayake, Manjusri Wickramasinghe

**Affiliations:** ^1^University of Colombo School of Computing, University of Colombo, Colombo, Sri Lanka; ^2^Faculty of Engineering and Design, University of Auckland, Auckland, New Zealand

**Keywords:** multi-agent systems, large language models, natural language processing, LLM agents, LLM-based MAS

## Abstract

**Introduction:**

Large Language Model-based Multi-Agent Systems (LLM-based MASs) represent a groundbreaking paradigm where diverse LLM-based agents collaborate, leveraging their unique capabilities to achieve shared objectives. Although LLM-based MASs outperform individual agents, their current architectures are limited by predefined, fixed, and static agent designs, restricting adaptability and scalability in dynamic environments.

**Method:**

To address these limitations, this study proposes two novel approaches: Initial Automatic Agent Generation (IAAG) and Dynamic Real-Time Agent Generation (DRTAG). These approaches enable the automatic creation and seamless integration of new agents into MASs, driven by evolving conversational and task-specific contexts, thereby reducing the need for human intervention. Our method leverages advanced prompt engineering techniques such as persona pattern prompting, chain prompting, and few-shot prompting to generate new agents through existing LLM agents. Additionally, several evaluation metrics were adapted to score and rank LLM-generated texts.

**Results:**

Experimental results demonstrate that the DRTAG approach significantly improves system adaptability and task performance compared to static MAS architectures. The IAAG framework also enhances initial system flexibility, supporting the creation of contextually relevant agents.

**Discussion:**

These findings highlight the potential of dynamic LLM-based MASs to overcome the limitations of static architectures to address complex real-world challenges, paving the way for innovative applications across diverse domains.

## 1 Introduction

Artificial Intelligence (AI) agents are autonomous entities designed to perceive their environment, remember situations, make decisions, and take actions. The concept of agents has philosophical roots with contributions from thinkers such as Aristotle and Hume (Schlosser et al., 2019). In AI, agents are seen as a step toward Artificial General Intelligence (AGI), capable of performing intelligent activities across various domains (Xi et al., 2025). Recent advances in Large Language Models (LLMs) highlight their potential as foundations for AGI, showcasing versatile abilities in knowledge acquisition, reasoning, and interaction.

Recently revolutionized LLMs, such as GPT-4 and BERT, have revealed remarkable performance in a wide spectrum of language tasks, setting novel standards in the field of Natural Language Processing (NLP) (Naveed et al., 2023; Llanes-Jurado et al., 2024; Eberhard et al., 2024; Baabdullah, 2024; Gao et al., 2024; Yan et al., 2024). Moreover, recent LLMs have emergent abilities to facilitate emergent language (EL), a field that investigates the form of communication that develops among artificial agents through interaction, without being explicitly pre-programmed (Peters et al., 2025; Wei et al., 2022). Hence, LLMs provide a strong foundation for both agent-to-agent and agent-to-human communications and can serve as the “brain” of AI agents, enabling them to adapt to various scenarios and perform complex tasks (Xi et al., 2025; Wei et al., 2022). AI agents with LLM brains (LLM-based agents) can be constructed by developing a framework that includes memory, perception, and action modules to allow them to interact with the environment in different ways.

The concept of LLM-based Multi-Agent Systems (MASs) involves a collective of diverse LLM-based agents, each possessing unique strengths, collaborating to achieve a common objective efficiently. This approach has shown significant improvements by leveraging the collaborative and coordinated efforts of multiple agents and tackling tasks that exceed the capabilities of any single agent (Guo et al., 2024). The growing popularity of LLM-based MASs is based on the success in complex problem-solving and world simulation. This paradigm shift highlights the potential of MASs to revolutionize various domains with more sophisticated and effective solutions.

Despite impressive advancements in LLM-based MASs, current frameworks still face significant limitations. One of the core challenges is the static nature of agent design, where most LLM-based MASs are predefined and not flexible enough to adapt to dynamic, evolving environments. In many cases, LLM agents in LLM-based MASs are manually crafted and require substantial human intervention to introduce new capabilities or modify existing ones. This restricts the scalability and real-time adaptability of LLM-based MASs, particularly in environments that demand continuous interaction, decision making, and problem-solving in real-time. Furthermore, while individual agents may excel at specific tasks, there is a lack of seamless integration methods to create new agents on the fly based on shifting conversational or task-specific requirements. These gaps highlight the need for an automated approach to agent generation and integration that can dynamically respond to environmental changes, thereby enhancing the overall efficiency and flexibility of LLM-based MASs.

This research addresses the evolving challenge of integrating AI agents into LLM-based MASs, a critical step toward achieving more adaptive and intelligent AI systems. In this study, we explore the dynamic integration of agents into LLM-based MASs to automatically scale MASs. We propose two novel one-shot approaches: Initial Automatic Agent Generation (IAAG) and Dynamic Real Time Agent Generation (DRTAG) approaches for the automatic generation and integration of LLM agents into an LLM-based MAS and evaluate these two novel approaches against one of the popular existing baseline approaches available in the literature for defining agents in LLM-based MASs at the time of this study. Our methods allow for real-time agent generation in response to evolving conversational contexts, significantly reducing the need for human intervention. The key contributions of this research are as follows.

1. **Application of prompt engineering techniques to utilize an LLM agent to create another LLM agent:** Introduction of advanced prompt engineering techniques such as persona pattern prompting, chain prompting, and few-shot prompting to facilitate dynamic LLM agent generation by using an existing LLM agent.
2. **Novel frameworks to automatically generate LLM agents for LLM-based MAS:** Introduction of IAAG and DRTAG approaches as novel frameworks for LLM-based MASs with the capability to automatically introduce new agents.
3. **Adaptation of several evaluation matrices to score and rank texts generated via LLMs:** Introduction of a systematic evaluation framework that adapts established text evaluation matrices in NLP domain to score and rank the quality and performance of LLM-generated texts within multi-agent systems when a similar external ground truth text is not available. In this evaluation, we use four complementary evaluation dimensions:

(a) **Binary weighting** for measuring task-related content coverage.
(b) **TF-IDF** (Term Frequency-Inverse Document Frequency) (Sparck Jones, 1972) for assessing the richness of relevant keywords to the topic.
(c) **MTLD** (Measure of Textual Lexical Diversity) (McCarthy and Jarvis, 2010) for evaluating lexical diversity and vocabulary richness.
(d) **BERTScore** (Bidirectional Encoder Representations from Transformers Score) (Zhang et al., 2019) for measuring thematic relevance and topical consistency.

Our experimental results demonstrate that the DRTAG method achieves significant improvements across all evaluation dimensions compared to baseline approaches. Specifically, DRTAG shows:

- Enhanced task-related content coverage as measured by binary weighting against task-specific keywords, indicating more comprehensive addressing of required task components.
- Improved task-related keyword richness score through TF-IDF analysis, demonstrating better utilization of domain-specific terminology and distinctive content generation.
- Increased lexical diversity scores via MTLD measurements, reflecting greater vocabulary variation and reduced repetition in generated text.
- Higher thematic relevance scores using BERTScore evaluation, confirming better thematic alignment and contextual understanding.

These quantitative improvements validate that DRTAG significantly enhances the richness of multi-agent conversations and the system's adaptability to complex task scenarios.

The remainder of this paper continues as follows. Initially, we set up the groundwork for our discussion in Section 2 by providing a brief overview of related work in the domain of LLM agents and LLM-based MASs. Section 3 elucidates the development process of our novel dynamic agent addition approach, along with an exploration of other existing dynamic agent approaches. In Section 4, we describe how we evaluated our proposed approaches against an existing approach and present the results of our experiments. Finally, we conclude our discussion with Section 5 offering an outlook on future work, respectively.

## 2 Related work

Recent advancements in LLMs have revolutionized NLP with their ability to approximate human-level performance on various tasks. They have evolved from statistical models to neural language modeling, and now to LLMs with billions of parameters that are capable of generalizing across multiple tasks (Naveed et al., 2023; Llanes-Jurado et al., 2024). LLMs have wide-ranging applications across various domains such as biomedical and healthcare, education (Oh et al., 2024), sports (Cook and Karakuş, 2024), social networks, business, engineering, and agriculture. They have the potential to solve real-world problems and shape the future of language understanding and generation (Raiaan et al., 2024). The transformer architecture (Vaswani et al., 2017), particularly the self-attention mechanism, has been pivotal in the advancement of LLMs. Variants like self-attention, cross-attention, and sparse attention contribute to their efficiency and effectiveness (Naveed et al., 2023; Raiaan et al., 2024).

### 2.1 Cutting edge LLMs and their variations

LLMs, such as Generative Pre-trained Transformer (GPT) models by OpenAI (Achiam et al., 2023), Gemini models by Google (Gemini Team et al., 2023), LLaMA models by Meta AI (Touvron et al., 2023), and the XLnet model (Yang et al., 2019) can be identified as cutting-edge AI models, specifically designed for NLP tasks. These LLMs, powered by transformer architecture, are trained on vast amounts of text data to comprehend and generate text with remarkable fluency and accuracy. Their proficiency in generating coherent text has been demonstrated in a wide range of tasks, including answering questions, summarizing texts, and translating languages (Naveed et al., 2023; Raiaan et al., 2024). Although popular LLMs such as GPT are trained using unlabelled data gathered from various sources such as Google, Wikipedia, and free books, there are several LLMs designed for specific tasks by fine-tuning the model with task-specific datasets (Raiaan et al., 2024). This process enhances their ability to follow user intent and generate aligned responses, improving zero-shot performance. Some popular biomedical task-specific LLMs are BioGPT (Luo et al., 2022) and BioBERT (Lee et al., 2020). However, we can limit models like GPT-3.5 and GPT-4 to generate responses for only a specific area of knowledge by using prompt engineering. All these popular LLMs and task-specialized LLMs are publicly available as APIs or online chatbots.

### 2.2 LLM-based agents

An autonomous version of GPT-4 called Auto-GPT has been designed to execute tasks with minimal human intervention (Firat and Kuleli, 2023). The core concept behind the Auto-GPT framework is AI agents that can self-manage, self-request commands, and execute tasks. This brought the concept of autonomous LLM agents to the field of computer science. The developers of Auto-GPT mentioned that integrating Auto-GPT into business and educational processes could enhance productivity and learning experiences. LLM agents like Auto-GPT are gradually improving with multi-step reasoning with complexity-based prompting (Fu et al., 2022) and self-consistency strategy (Wang et al., 2022). (Yang et al. 2023) introduced an algorithm called the Additional Opinion algorithm, which aims to significantly improve the performance of the online decision-making capabilities of LLM agents such as Auto-GPT. LLM agents can be utilized to manage existing AI models as well as other LLM agents. (Shen et al. 2024) have developed an agent called HuggingGPT that leverages LLMs to connect various AI models in machine learning communities such as Hugging Face to solve different tasks.

### 2.3 LLM-based MAS

A significant number of research endeavors are dedicated to the domain of Multi-Agent Systems (MAS), a computerized system that contains multiple autonomously interacting intelligent agents. These agents have their own unique abilities and they can work as a collaborative team to achieve a common goal in an efficient way. These agents can be software programs, robots, drones, Internet of Things (IoT) devices or a combination of those (Dorri et al., 2018; Mota et al., 2023; Jetly et al., 2012; Merdan et al., 2013; Fortino and and, 2013). Several recent research endeavors have focused on the implementation of MAS based on LLM by combining the argumentative and reasoning power of LLMs with collaborative interactions among intelligent agents to enable complex problem-solving and dynamic simulations. These LLM agents in MAS such as MetaGPT (Hong et al., 2023), Camel (Li et al., 2024), AutoGen (Wu et al., 2023), and AGENTVERSE (Chen et al., 2023) are able to break down tasks, explore information, and get advantage from each other, argue with each other (Ruiz-Dolz et al., 2025), and also compete with each other to progress the results beyond the capabilities of a single LLM agent's system. Multi-agent debate is an effective way to improve reasoning, adaptability, and factual accuracy in language models (Du et al., 2023; Talebirad and Nadiri, 2023). (Du et al. 2023) have proved that using multiple agents to generate solutions improves performance over using a single agent because multi-agent debate, which combines reflection (a language model critiques its own generation) and agent generation, gives a substantial boost in reasoning across all tasks.

### 2.4 Dynamic agent integration for LLM-based MAS

The prevalent approaches for LLM-based MASs rely on a prearranged set of agents that communicate with each other within a fixed architecture. Although research efforts conducted to develop popular LLM-based MASs such as AutoGen, MetaGPT, and CAMEL contributed significant advances to the LLM-based MAS domain, they still lack the capability to integrate dynamically generated agents into the MAS (Wu et al., 2023; Hong et al., 2023; Li et al., 2024). Designating specific roles for autonomous agents and recruiting new expert agents to form a group can augment the efficacy of MASs (Salewski et al., 2024). Hence, the inability to generate and integrate dynamic LLM agents into an LLM-based MAS constrains its adaptability to diverse tasks and necessitates strong human guidance in devising its agents (Liu et al., 2023).

**Figure 1:**
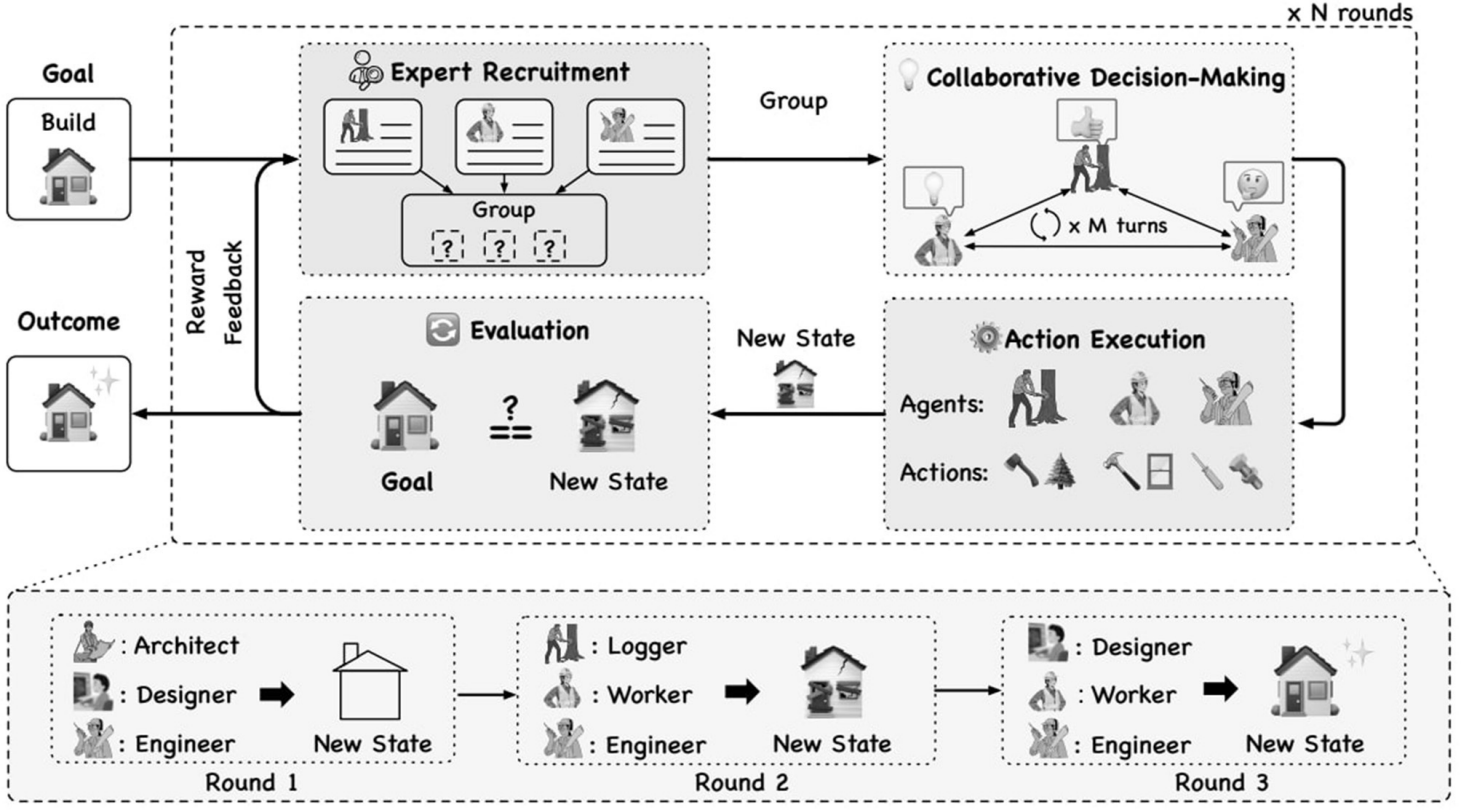
An illustration of the AGENTVERSE (Chen et al., 2023).

Few research endeavors mention about generating and integrating LLM agents dynamically into an LLM-based MAS. (Talebirad and Nadiri 2023) have developed a MAS where each agent has the ability to generate new LLM agents and the user can disable that ability for selected agents when initially defining the set of agents in the MAS. However, the underlying mechanism or the algorithm for dynamically generating and adding new agents has not been mentioned in that research work. (Chen et al. 2023) have developed a MAS called AGENTVERSE that has the ability to determine and adjust the agent group's composition based on the ongoing problem-solving progression. In the AGENTVERSE MAS, there's an agent called the recruiter that dynamically generates a set of expert descriptions based on the given task to generate a set of agents. The AGENTVERSE framework runs as an iterative process that evaluates the decisions taken by the agents at the end of each iteration and adjusts the composition of the agents based on the results of the evaluation for the next iteration, as shown in Figure 1. However, this proposed dynamic agent adjustment mechanism in the AGENTVERSE was not evaluated properly and this mechanism needs many iterations to identify the optimal set of agents for a given goal. Moreover, the dynamic agent adjustment mechanism in the AGENTVERSE was not a generalized approach and it was specifically designed for the AGENTVERSE framework.

Overall, existing approaches fail to offer robust and efficient methods for dynamically responding to changing tasks or conversational contexts in LLM-based MAS environments, limiting their potential for adaptive problem-solving. Hence, there's a clear research gap that underscores the critical need for generalized mechanisms and algorithms to enable real-time dynamic agent generation and integration within LLM-based MASs built using popular frameworks such as MetaGPT, CAMEL, and AutoGen.

While our research focuses specifically on one-shot dynamic agent generation approaches, we acknowledge that several other frameworks have explored different aspects of dynamic agent management. For instance, beyond AGENTVERSE, other systems have implemented various forms of agent adaptation and modification. However, our evaluation compares against the most directly comparable baseline approaches available in the literature at the time of this study, focusing specifically on frameworks that support one-shot agent generation rather than multi-iterative refinement processes. The comparison set includes the widely used AutoGen framework as a representative baseline, chosen for its accessibility and established performance in the field.

## 3 Proposed method

The aim of this research is to implement a robust one-shot framework to dynamically generate and integrate intelligent LLM agents into an LLM-based MAS. This study omits multi-shot agent-defining mechanisms such as the mechanism employed in the AGENTVERSE system (Chen et al., 2023) because defining the set of LLM agents in an LLM-based MAS by going through multiple iterations of concluded conversations produced by LLM-based MAS to refine and rearrange the set of participating LLM agents requires excessive temporal, computational, and financial resources when compared to one-shot mechanisms.

In this study, we identified and implemented the following three one-shot approaches to define the set of LLM agents in an LLM-based MAS.

1. **Letting the users define the set of LLM agents:** The first approach is to let the users define the set of LLM agents required for their specific context before initiating the conversation. This method is already implemented in Autogen, CAMEL, and MetaGPT frameworks (Wu et al., 2023; Li et al., 2024; Hong et al., 2023). Therefore, we reproduced this approach using the Autogen framework for evaluation purposes.
2. **Initial Automatic Agent Generation (IAAG):** This approach involves the preliminary generation and integration of LLM agents into the MAS automatically by the MAS itself based on the user input context.
3. **Dynamic Real Time Agent Generation (DRTAG):** This approach encompasses the real-time dynamic generation and integration of LLM agents, adapting responsively to the evolving conversational landscape within the MAS.

For the IAAG and DRTAG one-shot approaches, this research work introduces a novel LLM-based intelligent agent designed to function as the conversation manager with the capability of generating new LLM agents based on the content and requirements of the ongoing conversation among agents in an LLM-based MAS. This proposed conversation management agent is designed to suggest a name and a system prompt to create a new LLM agent by using the following prompt engineering techniques that have been demonstrated to enhance the quality of responses generated by LLMs in various literary works.

1. **Persona pattern prompting:** This technique involves crafting prompts that instruct the LLM agent to adopt a specific personality when responding. With this technique, the LLM agent can generate outputs that are tailored to a particular character or role, which can be useful for creating more engaging and contextually appropriate texts (White et al., 2023). In this proposed methodology, the persona pattern prompting technique is applied to assign a specific role for the proposed conversation management LLM agent. And we instruct the conversation management agent to apply this technique when generating a system prompt for a new LLM agent.
2. **Chain prompting:** This technique involves breaking a complex task into smaller, more manageable sub-tasks. By providing the LLM agent with step-by-step instructions, it can produce more coherent and comprehensive outputs. This approach is particularly effective for tasks that require a sequence of actions or a structured response (Sahoo et al., 2024). In this proposed methodology, the chain prompting technique is applied to mention what the conversation management LLM agent has to perform by using a simple sequence of tasks. And we instruct the conversation management agent as a sub-task to apply this technique when generating a system prompt for a new LLM agent.
3. **Few-shot prompting:** This technique involves giving a few examples to guide the LLM agent's behavior for a specific task, enabling it to quickly adapt and generate appropriate responses (Sahoo et al., 2024). In this proposed methodology, the few-shot prompting technique is applied to mention the currently available set of LLM agents in the MAS, their sample system prompts, and the latest snapshot of the ongoing conversation as examples for the conversation management LLM agent to generate names and system prompts for new LLM agents that are dynamically created and integrated to the LLM-based MAS.

In order to facilitate the few-shot prompting technique, the user has to define at least one LLM agent prior to starting the conversation in the novel IAAG and DRTAG approaches proposed in this research.

The proposed conversation management agent has the ability to determine the next LLM agent that subsequently participates in the conversation of the LLM-based MAS by using three distinct algorithms. Those algorithms are:

1. **Prompt engineering-based selection:** The conversation management agent analyses the current conversation context and the capabilities of existing LLM agents to determine the next most appropriate LLM agent for the continuation of the conversation by utilizing advanced prompt engineering techniques, specifically persona pattern prompting, chain prompting, and few-shot prompting.
2. **Round-robin algorithm:** This algorithmic approach cyclically selects the next LLM agent to provide its dialogue for the conversation, ensuring balanced participation across the MAS.
3. **Randomized selection:** An LLM agent is chosen at random from the available pool of LLM agents, introducing an element of unpredictability and diversity in the conversational flow.

**Figure 2:**
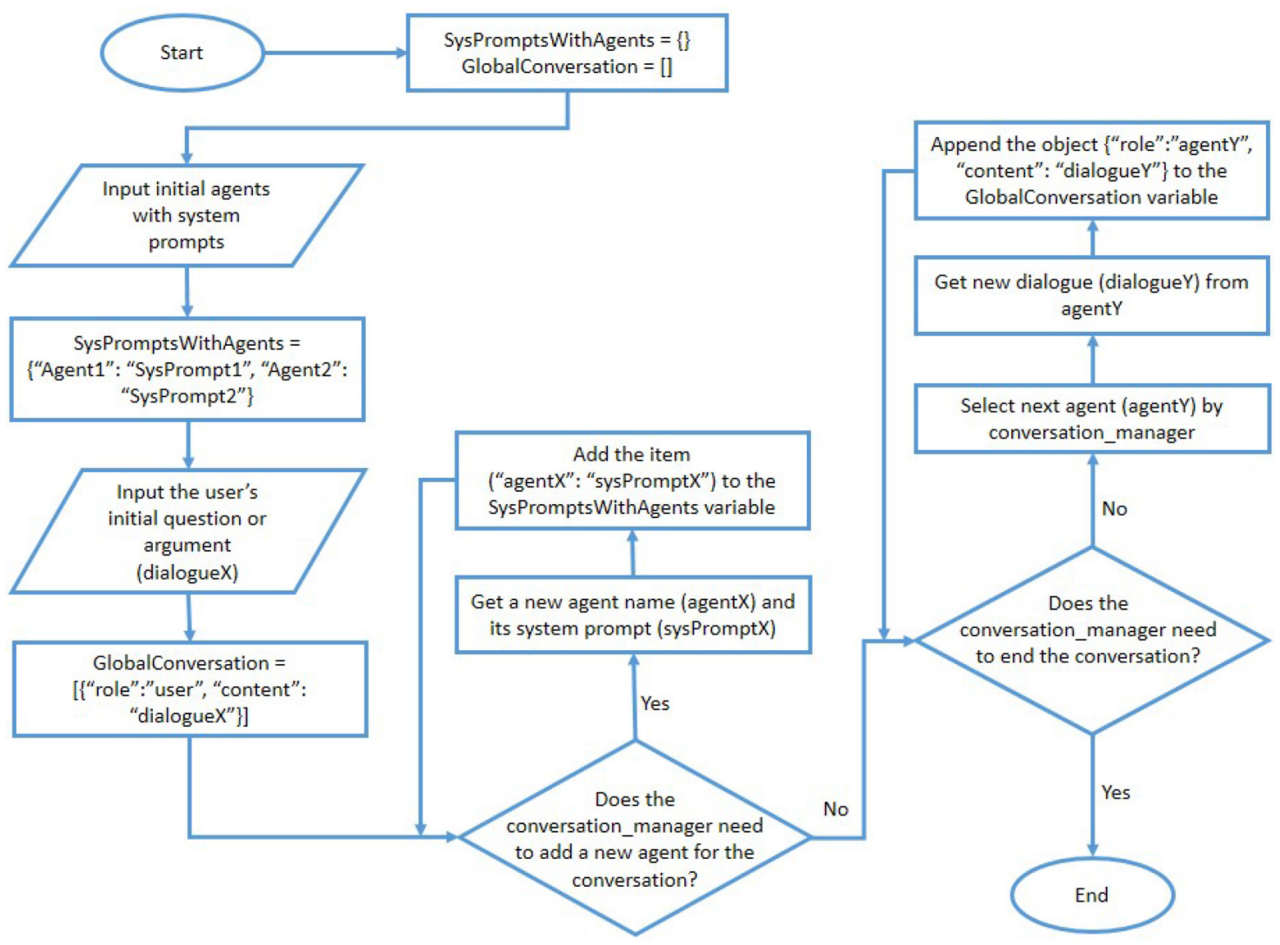
The flow chart for the proposed IAAG approach.

**Figure 3:**
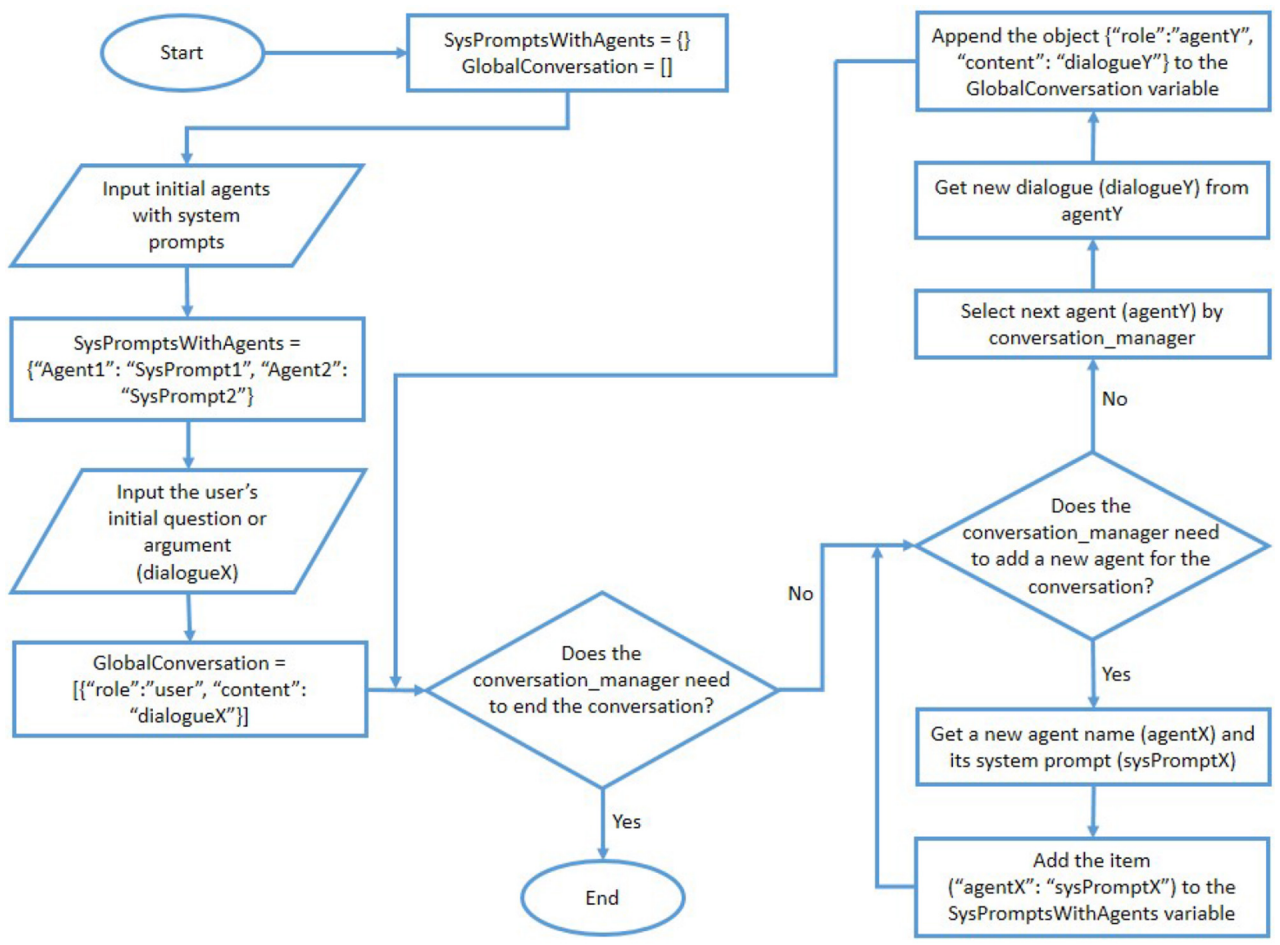
The flow chart for the proposed DRTAG approach.

The user of the proposed system can select one of the above three options to let the conversation management agent determine the order of agents' participation in the conversation. The overall process of the proposed IAAG approach is presented as a flow chart in Figure 2. Moreover, the overall process of the proposed DRTAG approach is presented as a flow chart in Figure 3.

## 4 Evaluation and results

As mentioned in Section 3, we implemented all three identified one-shot approaches for evaluation purposes. The first approach, which allows users to define the set of LLM agents, is reproduced using the Autogen framework, an existing state-of-the-art LLM-based MAS framework (Wu et al., 2023). The proposed novel approaches: IAAG and DRTAG were implemented using Python3 and the OpenAI Python library. As mentioned in Section 3, there are three agent selection algorithms that facilitate three distinct methods to determine the next LLM agent that subsequently participates in the MAS conversation. To ensure fair comparison and mitigate potential bias introduced by agent selection mechanisms to different approaches, all three approaches were integrated with the three distinct agent selection algorithms. Therefore, the following nine combinations of configurations were implemented in this study to evaluate the three identified one-shot approaches.

1. Autogen Framework with Prompt Engineering-Based (LLM-based) Selection.
2. Autogen Framework with Round-Robin Selection.
3. Autogen Framework with Randomized Selection.
4. IAAG Approach with Prompt Engineering-Based (LLM-based) Selection.
5. IAAG Approach with Round-Robin Selection.
6. IAAG Approach with Randomized Selection.
7. DRTAG Approach with Prompt Engineering-Based (LLM-based) Selection.
8. DRTAG Approach with Round-Robin Selection.
9. DRTAG Approach with Randomized Selection.

Only the GPT-4o model is used for generating the conversation through the above nine different implementations because employing the same LLM for all the above approaches will guarantee a fair evaluation of those approaches. A dataset with ten conversations from each of the nine configurations is generated to provide multiple opportunities for a single solution to surpass other approaches, thus enabling a comprehensive assessment of their productivity. Consequently, each one-shot approach: Autogen, IAAG, and DRTAG is represented by a total of thirty conversations in the final dataset, allowing for a robust and statistically meaningful comparison. This experimental design supports a rigorous quantitative analysis to determine whether either of the proposed novel approaches can statistically surpass the performance of the current state-of-the-art Autogen framework.

**Figure 4:**
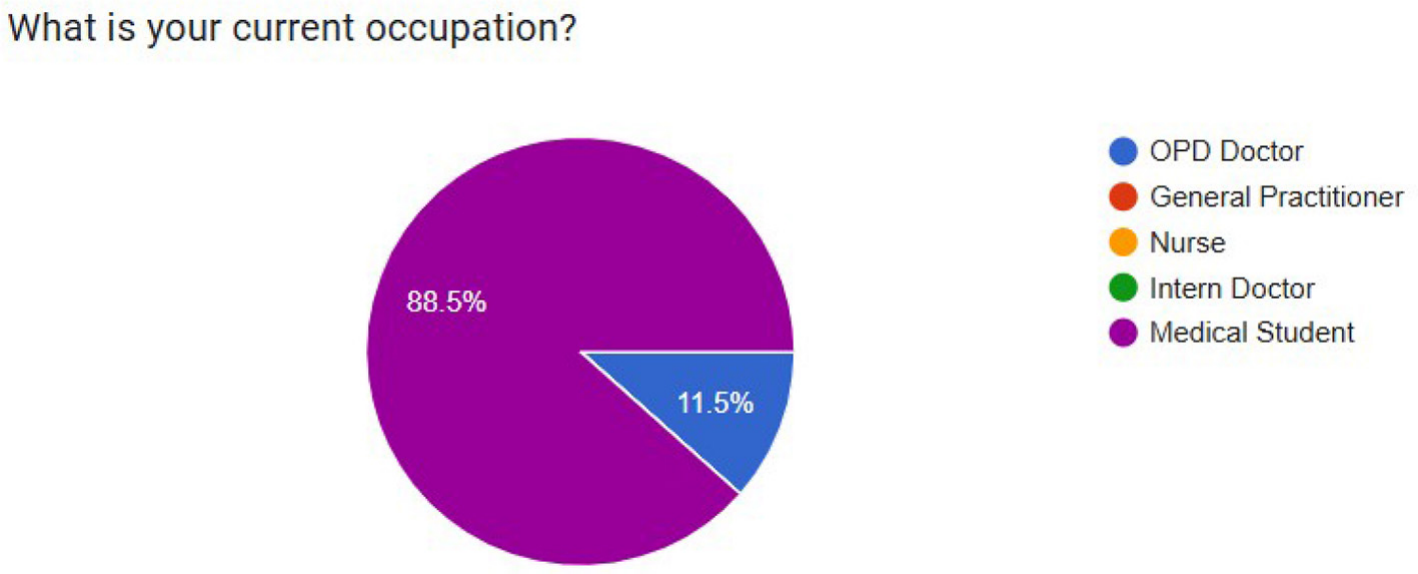
Summary of the occupational distribution of participants in the online questionnaire.

**Figure 5:**
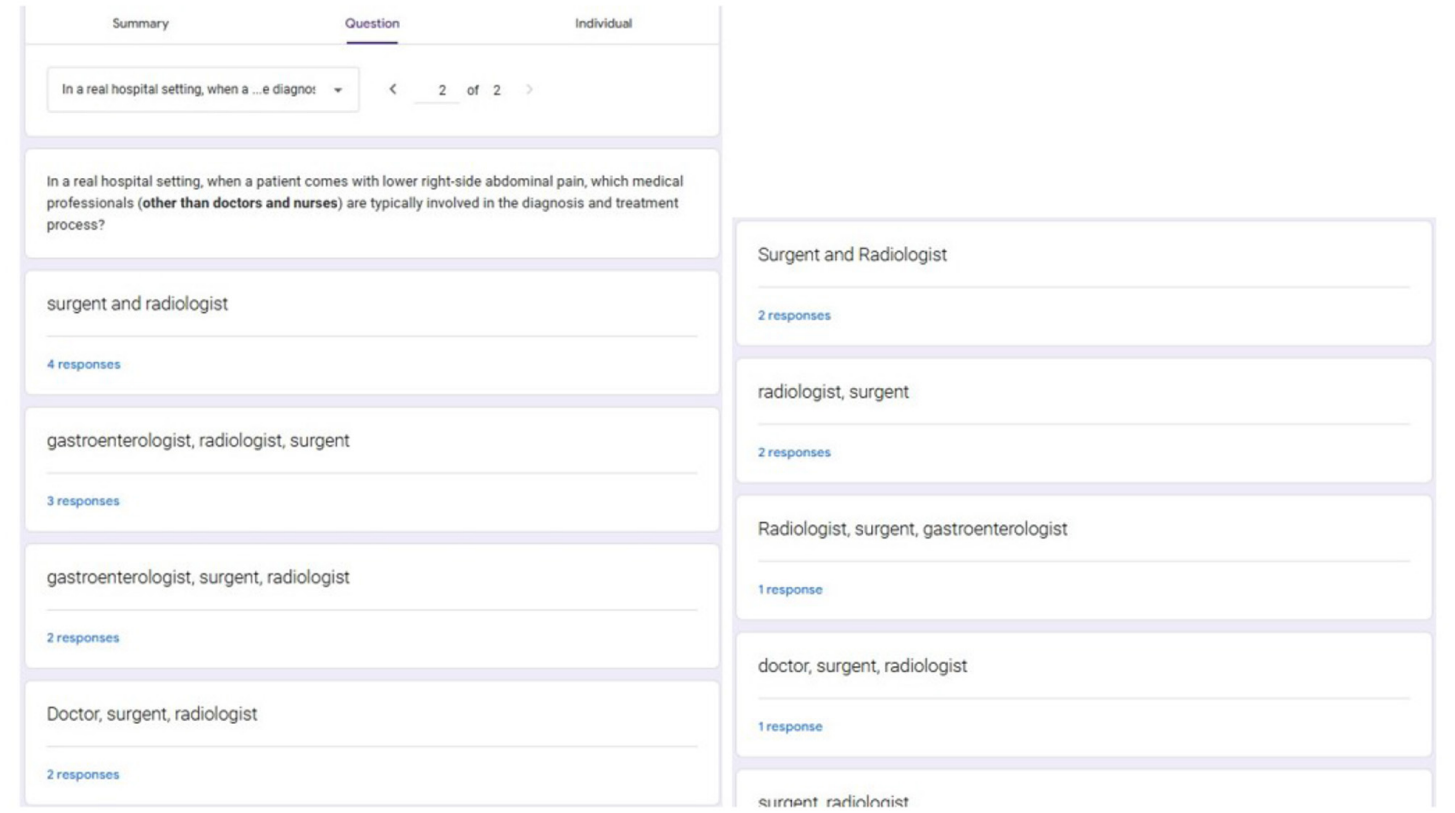
Sample short-answer responses provided by participants in the questionnaire.

To carefully assess these three approaches, we conducted a comprehensive evaluation by simulating a hospital environment with a situation in which a patient with lower right-sided abdominal pain comes to consult a doctor. This is a simple and common scenario, but it can encompass multifaceted aspects, leading to various inspections and diagnosis mechanisms for different illnesses with interactions among many different healthcare professionals. Therefore, this kind of example is ideal to evaluate an LLM-based MAS due to the scenario's complexity and realism and the environment's scalability and generalisability (Caruccio et al., 2024). The same medical scenario with the same user input was applied for all implementations to conduct a fair evaluation. As we mentioned in Section 3, we define two LLM agents: “Doctor” and “Nurse” in all nine implemented LLM-based MASs to facilitate the few-shot prompting technique. In addition to that, we defined three other LLM agents: “Radiologist,” “Surgeon,” and “Gastroenterologist” for MASs implemented by using the Autogen framework because existing LLM-based MAS solutions, such as Autogen and MetaGPT, are tailored for scenarios where users have prior knowledge of necessary participants and their roles. The decision to additionally pick these specific three LLM agents for the Autogen implementations is a result of a structured and anonymous questionnaire filled out by outpatient department (OPD) doctors and medical students. The questionnaire, designed as part of this research, has two questions: an MCQ question to identify the current occupation of the participant and a short answer question to identify medical professionals who are typically involved in the diagnosis and treatment process when a patient comes with lower right-sided abdominal pain. The results of the questionnaire are presented in Figures 4, 5. This evaluation design creates a challenging scenario for our novel approaches while highlighting their primary advantage: the ability to dynamically recognize when specialized expertise is required without prior domain knowledge. This approach better demonstrates the real-world applicability of our methods compared to frameworks like AutoGen that don't have the auto-scaling capability and require users to manually define all agents in advance.

**Figure 6:**
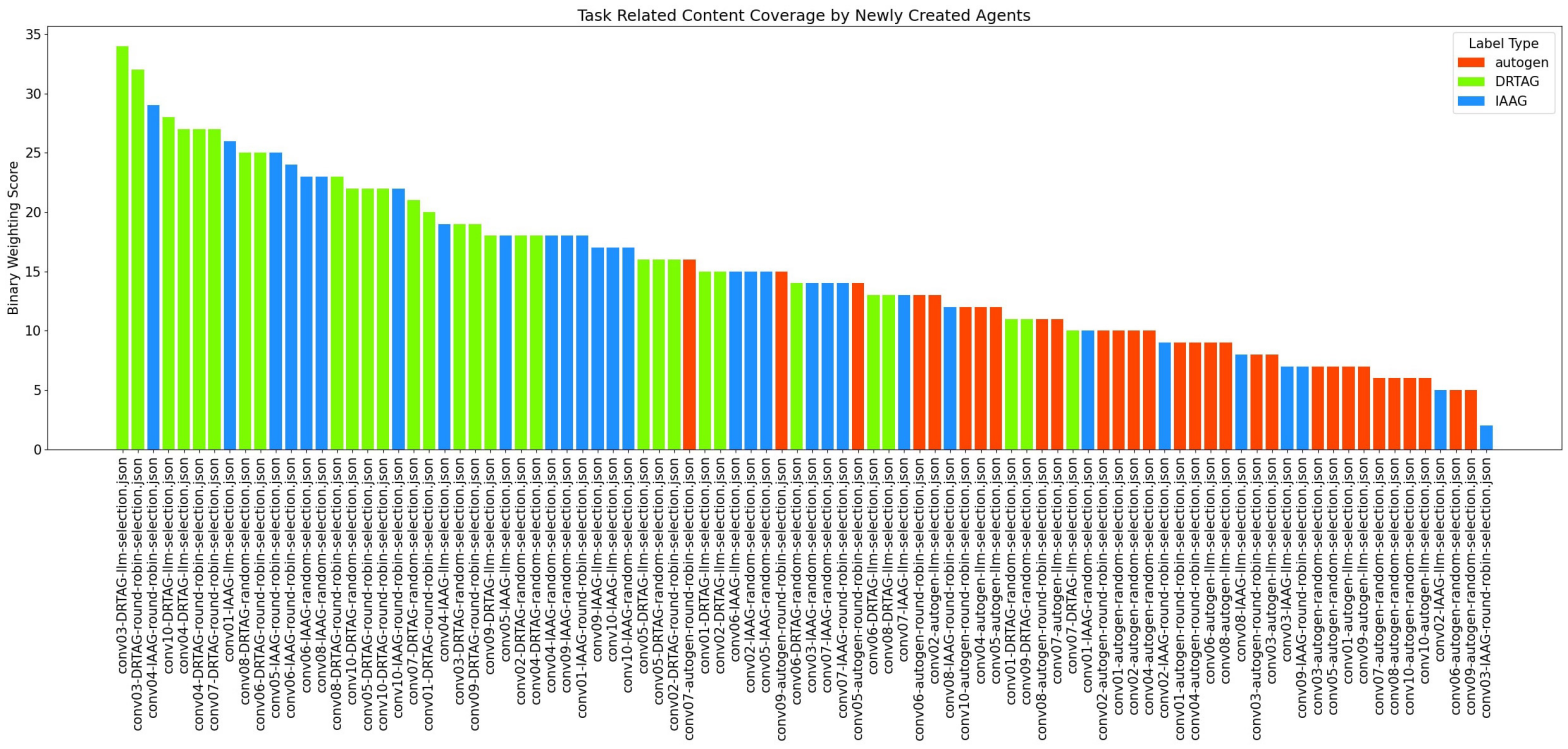
The bar graph that presents the task-related content coverage by newly introduced LLM agents to the MAS.

In this study, a conversation corpus (denoted as δ) is constructed by generating ten conversations from each of the nine different LLM-based MAS implementations. A set of keywords related to the given scenario is prepared by manually mining possible illnesses, diagnostic methods, proposed treatments, preventive actions, pre-treatment, and post-treatment advice from the corpus δ. Then, the set of mined keywords is generalized into unigram and bigram sequences of words to prepare a vocabulary of keywords (denoted as λ for scoring the conversations. Next, we scored each generated conversation in the dataset based on the following criteria:

1. **The task-related content coverage by newly introduced LLM agents:** The result scored with this criterion is shown in Figure 6. This is measured by binary weighting the relevant keywords produced by newly introduced agents to each conversation and conversation-wise adding those binary weights. In DRTAG and IAAG approaches, the agents automatically generated by the “conversation manager” agent are considered as “newly introduced agents”. In the Autogen framework, the agents suggested by participants of the online questionnaire are considered as “newly introduced agents”. If we take a random conversation *C*_1_ and denote the set of terms in *C*_1_ as α and set of terms generated by initial agents in *C*_1_ as β,

(1) $$\begin{array}{l} \text{Set of terms considered for binary weighting}C_{1}=\text{λ}\cap \left(\alpha -\beta \right) \end{array}$$

**Figure 7:**
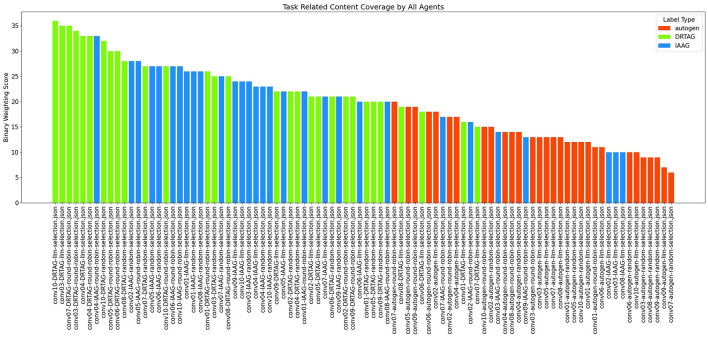
The bar graph that presents task-related content coverage by newly introduced LLM agents to the MAS, including the task-related content coverage by initial agents from their second dialogue.

**Figure 8:**
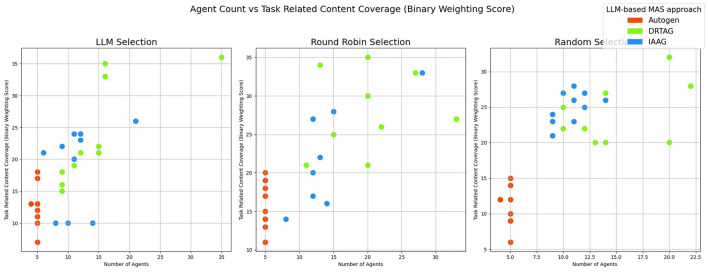
The scatter plot that presents the correlation between the number of agents participating in a conversation and the task-related content coverage of each conversation.

Since the newly generated agents can inspire the initial agents' dialogues, we scored the conversations again by considering β as the set of terms generated by initial agents only in their very first dialogue. The results of the second scoring approach are shown in Figure 7 and Figure 8.

**Figure 9:**
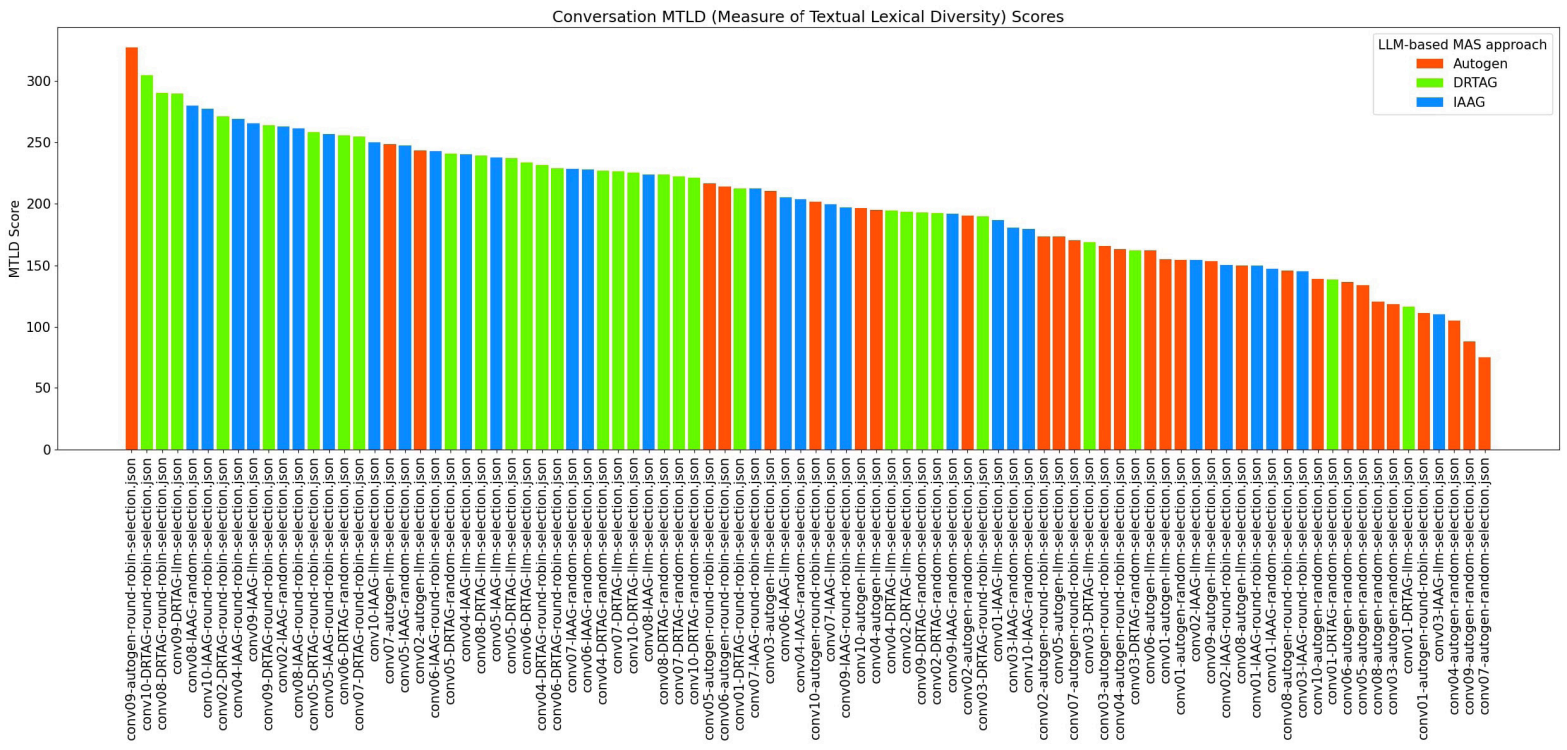
The bar graph that presents the lexical diversity (vocabulary richness) of each conversation in the corpus δ.

**Figure 10:**
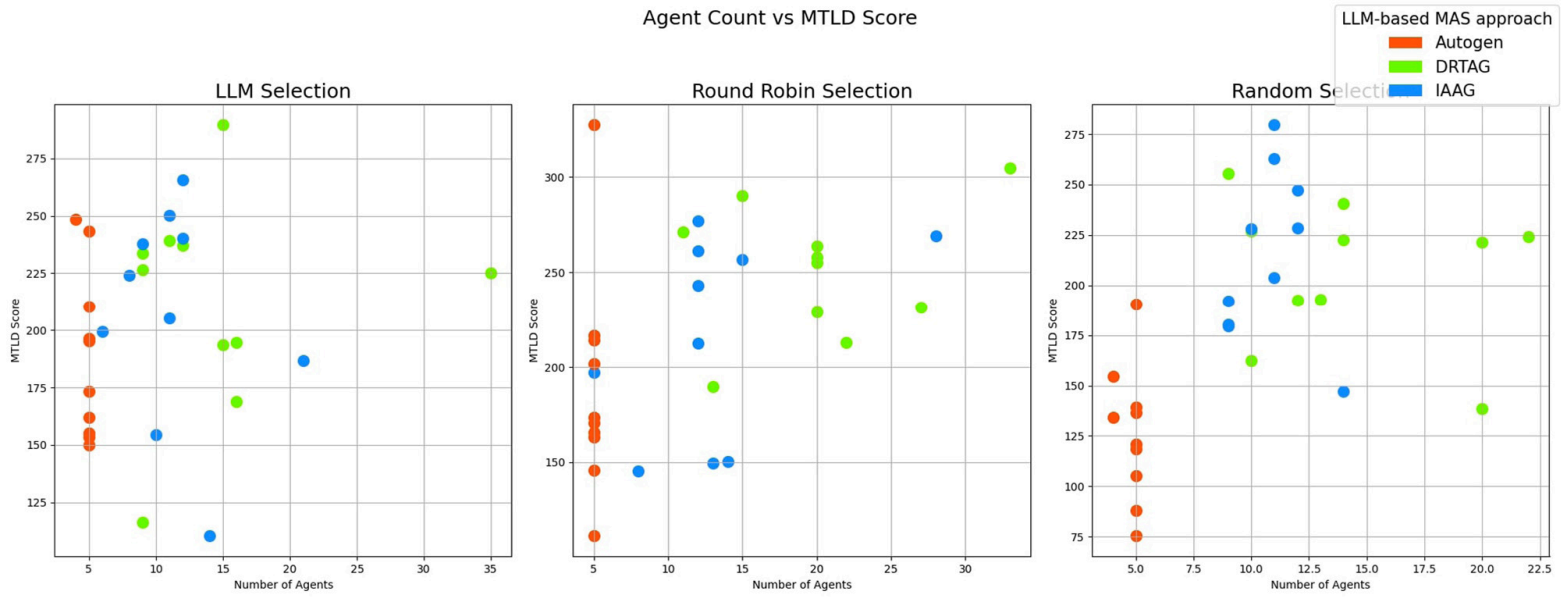
The scatter plot that presents the correlation between the number of agents participating in a conversation and the lexical diversity (vocabulary richness) of each conversation.

**Figure 11:**
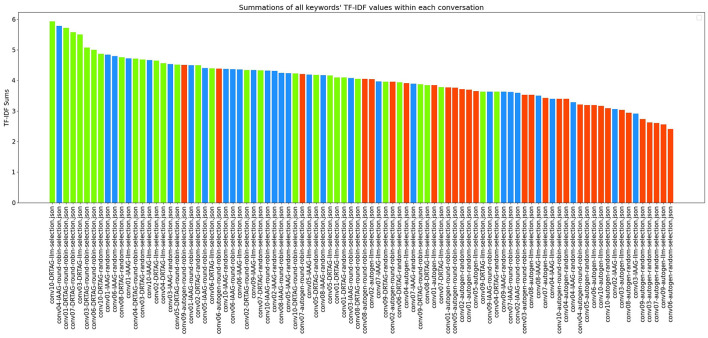
The bar graph that presents pairwise topical consistency of dialogues within the same conversation.

**Figure 12:**
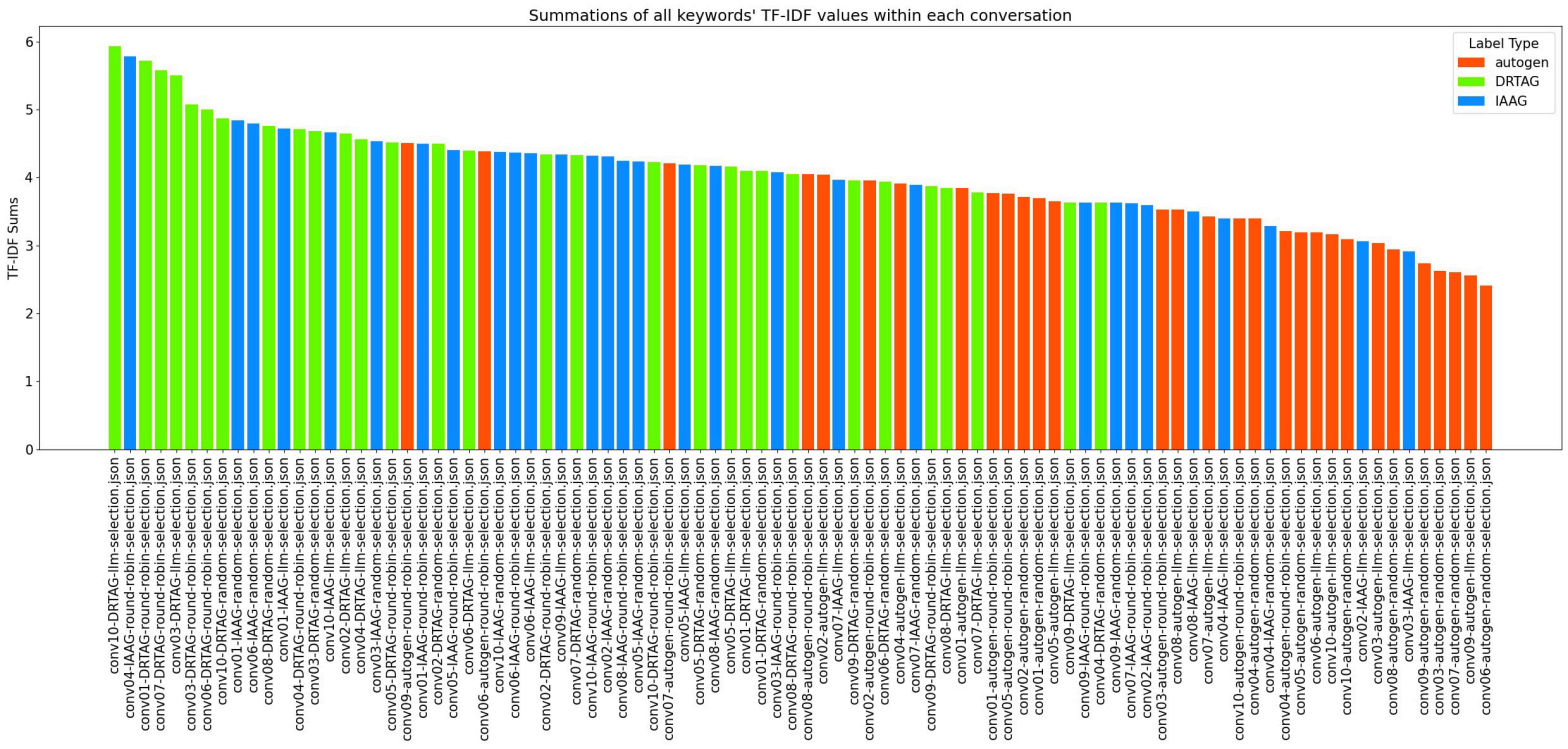
The bar graph that presents the richness of scenario-relevant keywords of each conversation in the corpus δ.

**Figure 13:**
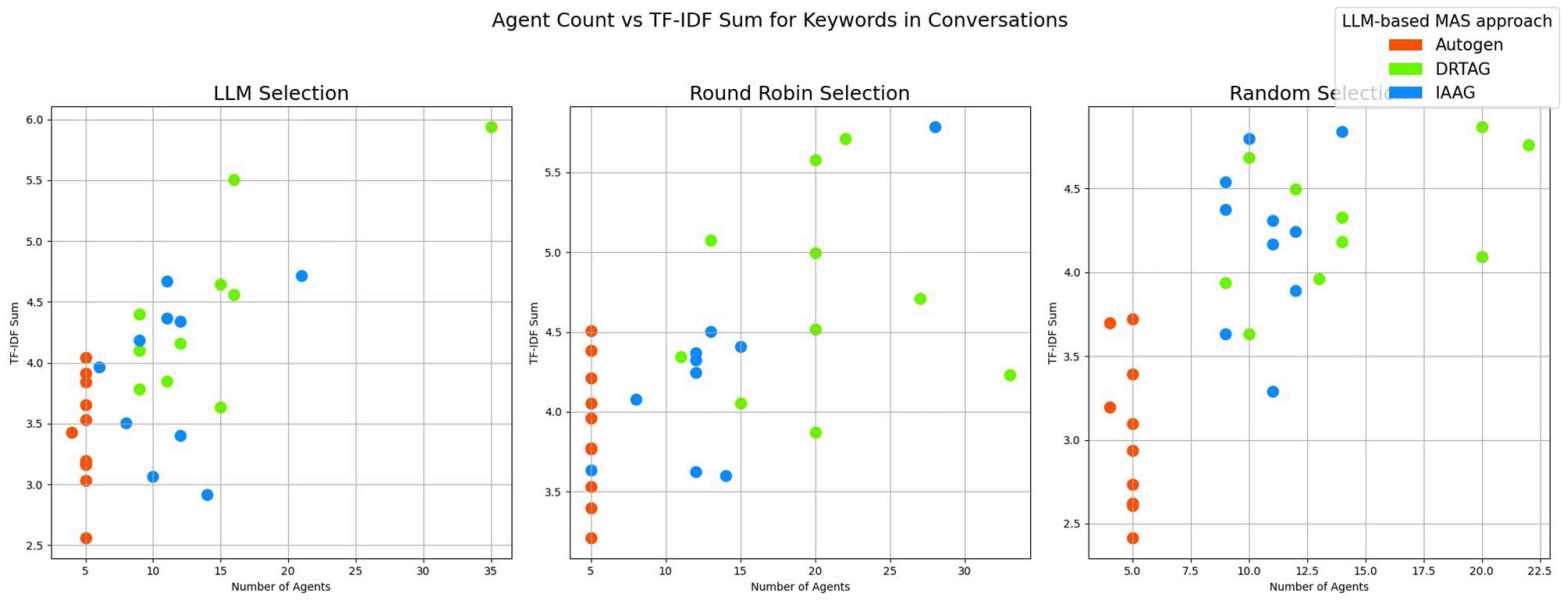
” The scatter plot that present the correlation between the number of agents participating in a conversation and the richness of scenario-relevant keywords of each conversation in the corpus δ.

2. **Lexical diversity and vocabulary richness of the conversation:** The results scored with this criteria is shown in Figure 9 and Figure 10. This is measured by using the MTLD scoring function in the Python lexical-diversity package.
3. **Pairwise topical consistency of dialogues within conversations:** This metric evaluates whether discussions explore topics with greater depth and breadth across individual dialogues, resulting in more diverse utterances. Topical exploration is quantified through pairwise BERTScore calculations between all dialogue pairs within conversations, where lower scores indicate enhanced topical depth and breadth exploration. Results are presented in Figure 11.
4. **The richness of relevant keywords:** To rank conversations in the corpus δ based on the richness of keywords in the vocabulary λ, we calculate the TF-IDF values of each keyword in λ for each conversation and rank conversations based on the TF-IDF values received by each conversation. Figure 12 shows the summations of TF-IDF values of each keyword in λ for each conversation in corpus δ and Figure 13 shows how the richness of relevant keywords of conversation depends on the number of agents in the MAS.

(2) $$\begin{array}{r} \text{Richness of relevant keywords of conversation}\\ C_{1}=\underset{t\in \text{λ}}{\sum }\text{TF-IDF}\left(t,C_{1}\right) \end{array}$$

**Figure 14:**
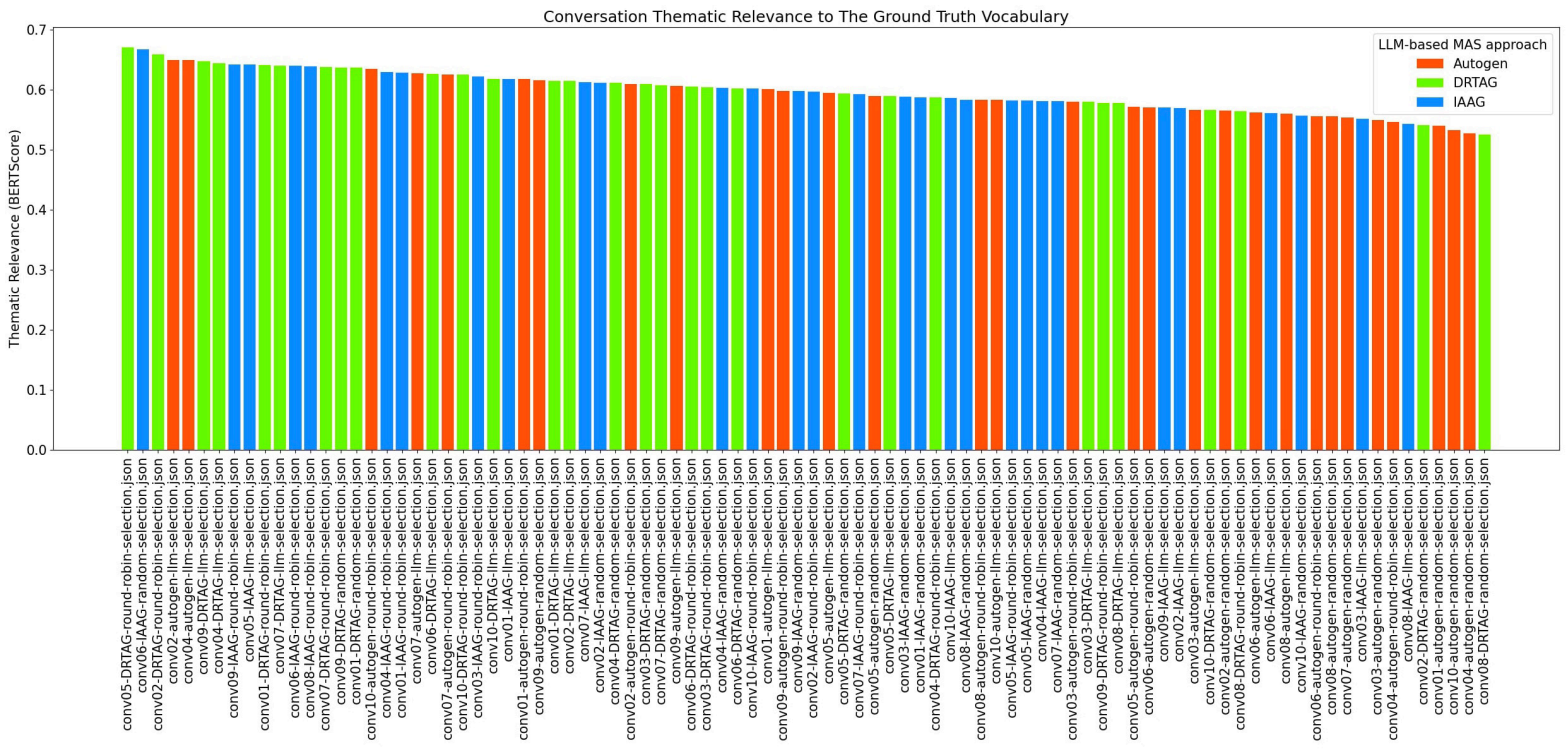
The bar graph that presents the thematic relevance of each conversation in the corpus δ calculated by using BERT embedding and cosine similarity.

5. **Thematic relevance of the conversation:** To rank the conversations in the corpus δ based on the thematic relevance, we calculated the BERTScore of each conversation against the vocabulary λ by calculating the cosine similarity between BERT embedded keywords in λ and BERT embedded conversations. The results of the BERTScoring are shown in Figure 14.

According to the results in Figure 6, dynamically generated agents using the DRTAG approach can cover more task-related content compared to carefully picked agents in Autogen implementations and the agents generated automatically by using the IAAG approach. Moreover, the results show that automatically generated agents in the IAAG approach can also cover more task-related content compared to carefully picked agents in Autogen implementations. We statistically measured these hypotheses by using the Mann-Whitney U Test (Mann and Whitney, 1947) to assess statistical significance and Cliff's Delta (Cliff, 1993) to measure the magnitude of differences across all comparisons. The comparison between DRTAG and Autogen yielded a p-value less than 0.0001, indicating that discussions generated by newly created agents in DRTAG contain significantly more keywords relevant to the scenario than those generated by Autogen agents. It also yielded a Cliff's Delta of 0.90, indicating a large effect size. Similarly, the comparison between IAAG and Autogen showed a p-value less than 0.0001 and a Cliff's Delta of 0.617, demonstrating that IAAG-generated discussions also contain significantly more scenario-relevant keywords than Autogen discussions with a large effect size. Furthermore, the direct comparison between DRTAG and IAAG revealed *p* = 0.0147, confirming that DRTAG agents generate discussions with significantly more task-relevant content than IAAG agents but with a small effect size indicated by a Cliff's Delta equal to 0.328. In all cases, we rejected the null hypothesis, providing strong statistical evidence that both proposed approaches (DRTAG and IAAG) outperform traditional Autogen implementations in generating task-relevant conversational content, with DRTAG demonstrating superior task-relevant content coverage over IAAG.

Figure 7 also shows similar results, but we observed some variations in the statistical significance levels. We can see similar results with both DRTAG and IAAG significantly outperforming Autogen, but the direct comparison between DRTAG and IAAG revealed *p* = 0.0948 and Cliff's δ = 0.198, which failed to reach statistical significance at the conventional level (0.05). In this case, we failed to reject the null hypothesis, indicating that there is no statistically significant evidence to conclude that discussions generated using DRTAG contain more scenario-relevant keywords than those generated using IAAG. By comparing binary weighting scores shown in Figures 6, 7, we can see that initially defined agents also explore new knowledge by mentioning new scenario-relevant keywords later with the influence of agents that are automatically generated through novel approaches because Figure 7 shows higher values than Figure 6 for many conversations. We checked whether there is a correlation between agent count and task-related content coverage by using Figure 8 and calculating the correlation coefficient (Pearson, 1909; Asuero et al., 2006) between agent count and binary weighting score. The result gave a correlation coefficient of 0.75, highlighting that there is a strong correlation between agent count and the task-related content coverage.

The results in Figure 9 show that the conversation's lexical diversity and vocabulary richness (MTLD score) are also high in many conversations generated via the DRTAG approach compared to conversations generated via the IAAG approach and Autogen framework. We also conducted Mann-Whitney U Tests to validate this hypothesis and found significant differences in lexical diversity between the approaches with a large effect size calculated with Cliff's Delta measure. The comparison between DRTAG and Autogen yielded a *p*-value less than 0.0001 and a Cliff's δ = 0.62, providing strong statistical evidence that discussions generated using DRTAG discuss the topic more broadly and deeply than discussions generated using Autogen. Similarly, the comparison between IAAG and Autogen showed *p* = 0.0004 and a large effect size with Cliff's δ = 0.504, demonstrating that discussions generated using IAAG also discuss the topic more broadly and deeply than discussions generated using Autogen. However, the direct comparison between DRTAG and IAAG revealed a *p*-value of 0.2506, which failed to reach statistical significance, and a Cliff's Delta of 0.102, which means that the effect size is negligible. These results suggest that while both novel approaches significantly enhance lexical diversity and vocabulary richness compared to the traditional Autogen framework, they demonstrate comparable conversational depth and breadth performance when directly compared to each other. We analyzed to determine whether there is a positive correlation between the number of LLM agents participating in each conversation and the lexical diversity and vocabulary richness of each conversation by using Figure 10 and a correlation coefficient measurement, which gave 0.42 as the result, indicating a weak correlation between agent count and MTLD score.

Then, we analyzed the correlation between the lexical diversity and the binary weight of the task-related keywords in a conversation, as these two metrics capture distinct yet potentially related aspects of conversational quality. The binary weighting of relevant keywords reflects the topical focus and task-specific content coverage by LLM agents, whereas lexical diversity (MTLD score) captures the variety and richness of the vocabulary used throughout the dialogue. Our analysis revealed a moderate positive correlation (Pearson's ρ = 0.58) between MTLD scores and binary weight scores. This indicates that conversations with higher topical relevance also tend to employ a richer and more varied vocabulary. In practical terms, the introduction of new agents that improve task coverage simultaneously enhances the linguistic diversity of the discourse. This finding indicates that these two aspects of conversational quality are not entirely independent, and improvements in one are likely to be accompanied by corresponding gains in the other.

A conversation can have a high lexical diversity, but it may repeat the same information with different words. To check this, we scored and ranked conversations by their pairwise topical consistency of dialogues within themselves by using BERTScore and the results are presented as a bar graph in Figure 11. The results in Figure 11 and our Mann-Whitney U Test also presented that both DRTAG and IAAG approaches demonstrate significantly lower topical consistency than Autogen, indicating greater conversational diversity. The comparison between DRTAG and Autogen yielded *p* = 0.0163 and Cliff's δ = –0.322, indicating a statistically significant but small effect size. This provides statistical evidence that discussions generated using DRTAG have lower topical consistency than those generated using Autogen, suggesting that while DRTAG discussions tend to explore the topic more deeply and broadly within each dialogue, the observed difference in topical consistency is small in magnitude. Similarly, the comparison between IAAG and Autogen showed *p* = 0.0242 and Cliff's δ = –0.298, demonstrating that discussions generated using IAAG also have lower topical consistency than discussions generated using Autogen but with small magnitude in differences. However, the direct comparison between DRTAG and IAAG revealed no significant difference (*p* = 0.3476), indicating comparable performance in avoiding repetitive information while both approaches outperform Autogen in generating topically diverse conversations.

The results of our TF-IDF evaluation to determine the richness of relevant keywords are presented in Figure 12 as a bar graph. The bar graph clearly shows that conversations generated via the DRTAG and IAAG approaches are richer in task-related keywords than conversations generated via the Autogen framework. We tested this hypothesis by applying Mann-Whitney U Tests, and the results show that conversations generated via the DRTAG approach are richer in task-related keywords than conversations generated via the Autogen framework, with a p-value less than 0.0001. We also calculated Cliff's delta to measure the effect size, which yielded a value of 0.818, indicating a large effect size. Similarly, the statistical comparison between IAAG and Autogen showed *p* = 0.0001 and Cliff's δ = 0.582, demonstrating that discussions generated using IAAG also utilize domain-specific terminologies more than discussions generated using Autogen, with a large effect size. Furthermore, the direct comparison between DRTAG and IAAG revealed *p* = 0.0307, confirming that both proposed approaches significantly outperform the traditional Autogen framework in generating conversations rich with task-relevant terminology, with DRTAG demonstrating superior performance over IAAG in utilizing domain-specific vocabulary, but with a small effect size, indicated by Cliff's δ = 0.282. With Figure 13 and the resulting correlation coefficient we calculated, there is a strong positive correlation between agent count and TF-IDF summations, indicating the richness of relevant keywords of the conversation. Therefore, dynamically auto-scaling the number of agents in MAS's conversations leads to enhanced utilization of domain-specific terminology and task-relevant vocabulary, suggesting that larger agent ensembles contribute to more comprehensive and contextually rich discussions within the problem domain.

Calculating only the TF-IDF against keywords in the vocabulary λ will give us insights into the frequency and importance of domain-specific terms within conversations, but there can be a set of other n-grams that contextually align with the ground truth. Therefore, we calculated the thematic relevance of each conversation against the vocabulary λ by using BERTScoring to evaluate the semantic similarity and contextual alignment between the generated discussions and the target, providing a more comprehensive assessment of conversations' thematic relevance. Figure 14 shows the results of our thematic evaluation, and it clearly shows that most conversations, generated via DRTAG and IAAG approaches, are more thematically related than conversations generated via the Autogen framework. This hypothesis was also tested by applying Mann-Whitney U Tests, and the result proved that conversations generated via the DRTAG approach are more thematically relevant to the scenario than conversations generated via the Autogen framework, with a *p*-value of 0.0047 and a medium effect size (Cliff's δ = 0.391). Similarly, conversations generated via the IAAG approach are also more thematically relevant to the scenario than conversations generated via the Autogen framework, with a *p*-value of 0.0425 and a small effect size (Cliff's δ = 0.260). However, the direct comparison between DRTAG and IAAG revealed no statistically significant difference with a *p-*value of 0.1031, indicating that both approaches demonstrate comparable performance in generating thematically relevant conversations. At the same time, both significantly outperform the traditional Autogen framework in semantic alignment with the target domain.

**Figure 15:**
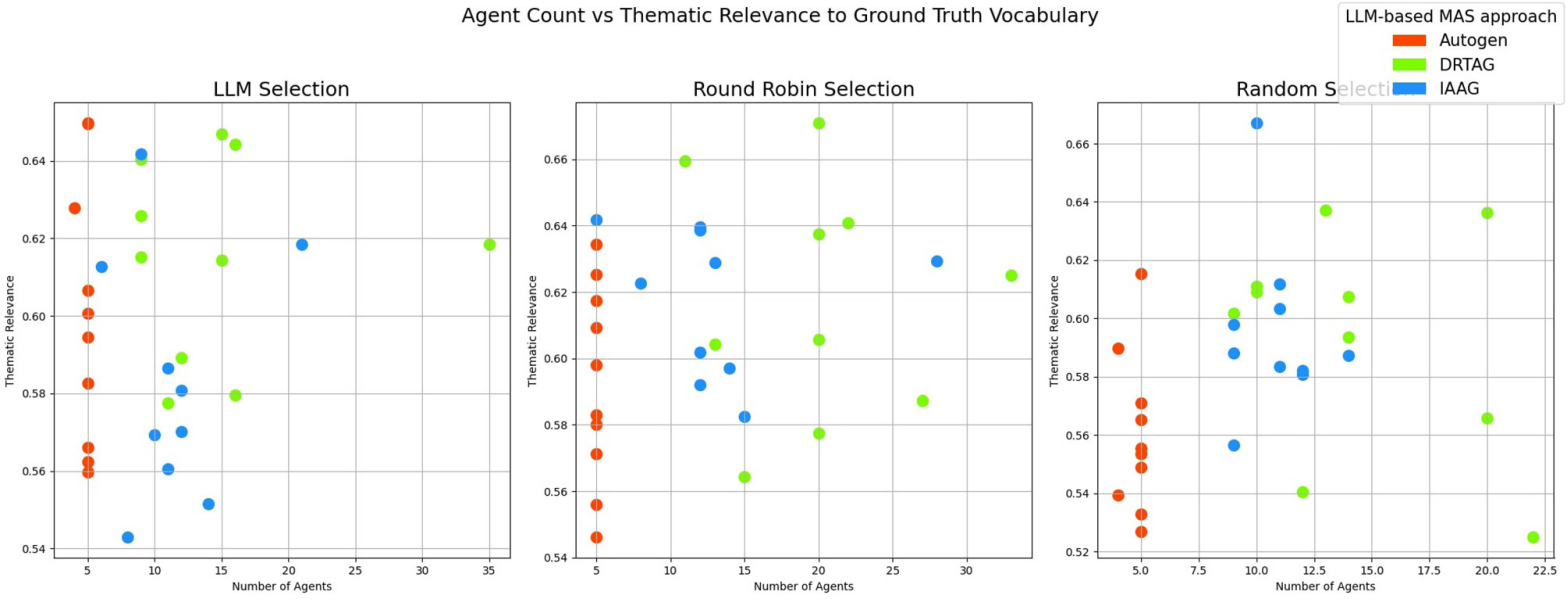
The scatter plot that presents the correlation between the number of agents participating in a conversation and the thematic relevance of each conversation in the corpus δ.

Then, we verified whether there is a positive correlation between the number of agents who participated in the conversation and the thematic relevance of the conversation. However, the result ended up with a 0.22 correlation coefficient that shows a very weak correlation, and the summary of the results is presented as a scatter plot in Figure 15.

Finally, we further examined the relationship between the thematic relevance of conversations and the richness of relevant keywords, measured by the summation of TF-IDF values of the domain-specific vocabulary. Thematic relevance, computed via BERTScore against the domain-specific vocabulary, reflects the semantic alignment of the conversation with the target domain, while the TF-IDF sum captures the extent and distinctiveness of keyword usage within the conversation. Our analysis revealed a weak positive correlation (Pearson's ρ = 0.34) between these two measures. This result indicates that conversations with higher thematic relevance also exhibit slightly greater richness in domain-specific keyword usage, but the relationship is not strong. This result indicates that LLM-based MAS can generate semantically well-aligned conversations with the domain without relying heavily on the explicit use of distinctive domain-specific keywords.

## 5 Conclusion

In this research, we presented a comprehensive exploration of dynamic agent integration within LLM-based Multi-Agent Systems (MAS) while identifying a critical gap in existing LLM-based MAS frameworks, which is the inability to dynamically generate and integrate LLM agents into the MAS in order to facilitate the evolving environment. As a solution to fill the identified gap, this study introduced two novel one-shot approaches to auto-scale the LLM-based MAS by automatically introducing a set of LLM agents. The proposed novel approaches are named as Initial Automatic Agent Generation (IAAG) and Dynamic Real Time Agent Generation (DRTAG). These approaches were meticulously designed to enhance the adaptability and efficiency of LLM-based MAS by enabling the real-time generation and integration of agents in response to the evolving conversational context.

In existing solutions, such as the Autogen framework, users must initially select and meticulously define a set of agents to achieve optimal results. This process necessitates users' substantial domain knowledge to identify the most essential agents for a given scenario. However, the proposed novel approaches have successfully mitigated this requirement by automatically generating all the necessary agents for the environment and the given scenario with the help of a predefined conversation management agent. The proposed conversation management agent leverages advanced prompt engineering techniques, such as persona pattern prompting, chain prompting, and few-shot prompting to facilitate the dynamic generation of new agents. These techniques ensure that the MAS can adapt to complex tasks and scenarios without extensive human intervention.

The evaluation, conducted by using the corpus that contains ninety conversations generated by using nine different LLM-based MAS configurations and a simulated medical scenario, demonstrated the superiority of the proposed DRTAG approach in enriching conversations. The experimental results reveal that DRTAG achieves significant improvements across all evaluation dimensions compared to the baseline approach. Specifically, DRTAG exhibits enhanced task-related content coverage as measured by binary weighting against task-specific keywords, indicating more comprehensive addressing of required task components. The approach also demonstrates improved task-related keyword richness scores through TF-IDF analysis, reflecting better utilization of domain-specific terminology and distinctive content generation. Additionally, DRTAG shows increased lexical diversity scores via MTLD measurements, indicating greater vocabulary variation and reduced repetition in generated text, alongside higher thematic relevance scores using BERTScore evaluation, which confirms better thematic alignment and contextual understanding. These quantitative improvements validate that auto-scaling the LLM-based MAS by using the DRTAG approach significantly enhances the richness of multi-agent conversations and demonstrates superior system adaptability to complex task scenarios.

Overall, the research contributes to addressing limitations in the field of LLM-based MAS by providing generalized mechanisms for dynamic agent integration, building upon existing foundations in the field. These findings pave the way for future advancements in the development of intelligent and autonomous LLM-based MAS since these proposed DRTAG and IAAG methods have generalized algorithms to facilitate seamless integration with existing systems, such as AutoGen and MetaGPT. The proposed methods hold great promise for a wide range of applications, from healthcare to education, where the ability to dynamically adapt to new information and tasks is crucial.

As a future work, the proposed approaches and existing approaches can be evaluated against several other medical scenarios as well as some other scenarios related to other industries such as law, engineering, music, education, management, etc. In the current study, we conducted a statistical evaluation and comparison of only the baseline approach and the two proposed novel methods. However, a more comprehensive analysis involving all nine possible configurations, comprising three approaches (Baseline, DRTAG, and IAAG) combined with three selection algorithms (LLM selection, random selection, and round-robin selection) remains an open area for exploration. Future research could also incorporate domain expert evaluations to assess and rank the quality of the generated corpora, thereby strengthening the reliability and validity of the results. Future researchers can put an effort into improving the prompts, including system prompts engineered to utilize the “Conversation Manager” agent as an LLM agent creator in order to improve the output of these proposed approaches. As a future work, utilizing explainable AI techniques to extract faithful and efficient global explanations for the original LLM over multiple tasks can lead to even greater performance gains, making these systems even more capable of reliably handling complex real-world challenges autonomously (Agiollo et al., 2024).

## Data Availability

The datasets presented in this study can be found in online repositories. The names of the repository/repositories and accession number(s) can be found below: https://github.com/ravinduramesh/Dynamic-Agent-Intro-Multi-Agent-LLM-Systems.

## Appendix A

### Python code for implementations and evaluations

The complete code for the implementations of the existing solutions and the noval approaches can be found in a Github Repository. This Github repository contains all the evaluation scripts with the results that we used for evaluate the implementations in this research.
